# Food and drug administration’s critical path initiative and innovations in drug development paradigm: Challenges, progress, and controversies

**DOI:** 10.4103/0975-7406.72130

**Published:** 2010

**Authors:** Rajiv Mahajan, Kapil Gupta

**Affiliations:** Department of Pharmacology, Adesh Institute of Medical Sciences and Research, Bathinda - 151 109, India; 1 Department of Biochemistry, Adesh Institute of Medical Sciences and Research, Bathinda - 151 109, India

**Keywords:** Biomarkers, clinical trials, drug development, microdosing

## Abstract

During the last decade, despite increased investment in drug research and development related activity, stagnation in new drug discovery has been documented. Despite a 70% increase in investment in research and development-related activities, a 40% fall in launch of new chemical entities was seen during 1994–2004. A steep rise in the attrition rate of drug development has complicated the matter. Rising cost and increased attrition rates proved major barriers to investment in higher risk drugs or in therapies for uncommon diseases or diseases that predominantly afflict the poor. This prompted Food and Drug Administration (FDA) to highlight this problem in a 2004 white paper classified as “Critical Path Initiative” (CPI) and to initiate steps to target stagnation and rise in attrition rates. Many new drug development projects have started worldwide taking cue from CPI; adopting microdosing, adaptive designs and taking advantage of newly developed biomarkers under the CPI. This review discusses the various strategies adopted under CPI to decrease attrition rate and stagnation of new drug development, and the challenges and controversies associated with CPI.

New drug development is a complex, long and expensive activity. The cost of development of one successful marketed drug is estimated to range from US$ 802 million to US$ 1 billion.[1,2] The average time required for the development of a new drug is about 9 years.[3] During the last decade, attrition rates have also increased. A new medical compound entering phase I testing, after years of preclinical screening and evaluation, is estimated to have only an 8% chance of reaching the market.[4] Moreover, only 2 of 10 marketed drugs return their research and development (R and D) investments.[5] To make the matter worse, stagnation in the development of new drug entities has been documented, despite increased spending on R and D related activities compared to previous decades.[6]

Rising cost, increased attrition rates and risk of non-recovery of investments incurred on drug development are the major barriers to investment in innovative, higher risk drugs or in therapies for uncommon diseases or diseases that predominantly afflict the poor. So, researchers across the globe have shown interest in applying better designs to expedite the approval of potential medicinal products, so as to cut cost and time-expenditure on new drug development without compromising efficacy and safety. Accordingly, in 2004, United States Food and Drug Administration (FDA) released a white paper, now known as the Critical Path Initiative (CPI), thus calling attention to an alarming decline in the number of innovative medical products being submitted for FDA approval.[4] In 2006, the FDA released a Critical Path Opportunities List that calls for better evaluation tools, streamlining clinical trials, and developing approaches to address urgent public health needs.[7] Subsequently, FDA reports, highlighting critical path activities undertaken during previous years were released.[8,9] The latest update on CPI was released in March 2010.[10]

## Historical Background

From the beginning of the civilization, people have been concerned about the quality and safety of foods and medicines. In 1202, King John of England proclaimed the first English food law, the Assize of Bread, which prohibited adulteration of bread with ground peas and beans. Regulation of food in the United States dates from early colonial times. Federal controls over the drug supply began with inspection of imported drugs in 1848, although the first federal biologics law, which addressed the provision of reliable smallpox vaccine to citizens, was passed in 1813.[11] In 1937, sulfanilamide tragedy struck, thus mandating the need to establish drug safety before marketing the new drug. Subsequently, The Federal Food, Drug, and Cosmetic (FDC) Act of 1938 was passed, thus extending control to cosmetics and therapeutic devices and requiring new drugs to be shown safe before marketing; starting a new system of drug regulation.[12]

In 1949, FDA published guidance to industry for the first time. This guidance, “Procedures for the Appraisal of the Toxicity of Chemicals in Food,” came to be known as the “black book”.[13] In 1962, thalidomide, a new sleeping pill was found to have caused birth defects in thousands of babies born in western Europe. News reports on the role of Dr. Frances Kelsey, FDA medical officer, in keeping the drug off the US market, arouse public support for stronger drug regulation. Accordingly, Kefauver-Harris Drug Amendments were passed in 1962 itself to ensure drug efficacy and greater drug safety. For the first time, drug manufacturers were required to prove to the FDA the effectiveness of their products before marketing them.[14] In 1976, Medical Device Amendments were passed to ensure safety and effectiveness of medical devices, including diagnostic products. In 1991, FDA published regulations to accelerate the review of drugs for life-threatening diseases.[15]

**Figure 1:**
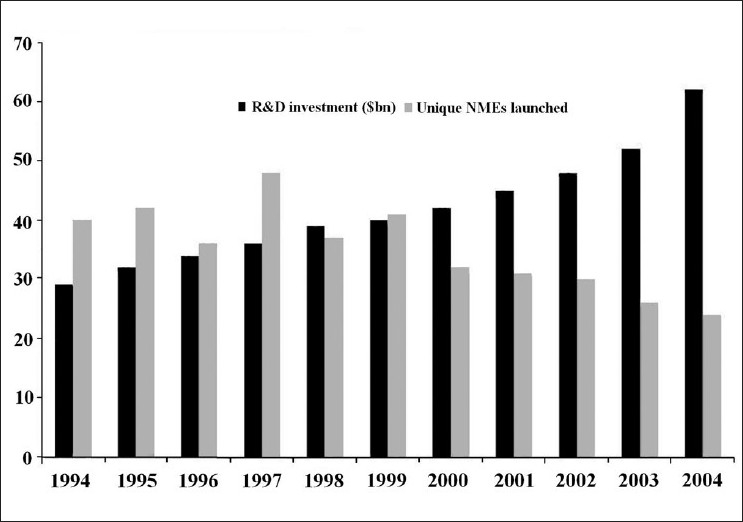
Comparison between R and D investment and NMEs launched between 1994 and 2004[15]

From 1998, progressive stagnation in the number of applications submitted to FDA for new medical entities was observed, despite a rise in investment in R and D. It was estimated that global R and D investments had risen by 70% from 1994 to 2004, while the output of new molecular entities (NME) had fallen by 40%, during the same period[15] [Figure 1].

The problem has been compounded with the rising attrition rate. In the past decade, attrition was highest (62%) during phase II. It was 45% in phase III and 23% at the time of registration.[16] A 1991 study showed 32% failure rates in phase I and 75% phase transitional probability from phase I to phase II,[17] while a later study conducted in 2003 by the same authors showed failure rates in phase I to increase to 37% and phase transitional probability from phase I to phase II to decrease to 71%, over a period of 12 years.[1]

A 2004 white paper of FDA documented that the investment required to launch a new drug in the market had risen by 55%; from $1.1 billion to $1.7 billion, during 2000–2002 as compared to 1995–2000. This paper also highlighted that there was stagnation in the submission of applications for new chemical entities. It was stated that current stagnation was because the prevalent medical product development path was becoming increasingly challenging, inefficient, and costly. Another reason cited was that very often, developers were forced to use the tools of the last century to evaluate this century’s advances. It was felt that new product development toolkit, containing powerful new scientific and technical methods such as animal or computer-based predictive models, biomarkers for safety and effectiveness, and new clinical evaluation techniques, was urgently needed to improve predictability and efficiency along the critical path from laboratory concept to commercial product.[4]

## Critical Path and Critical Path Initiative

**Figure 2:**
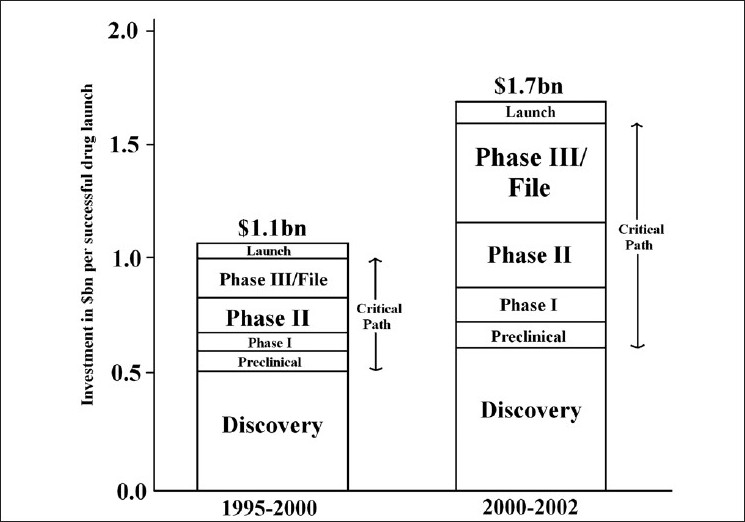
Comparative investment on new successful drug launch between 1995–2000 and 2000”2002[4]

Time incurred in drug development after drug discovery, i.e., from the time of beginning of preclinical studies to the launch of a drug in the market is stated to be “Critical Path” and it is estimated that most of the recent investment increases are within the critical path development phase; cost during discovery phase remain more or less the same[4] [Figure 2].

Thus, innovative methods were planned and initiated to target this critical path, so as to bring down cost of development, rate of attrition and stagnation and this initiative was called CPI. The CPI is FDA’s policy document to drive innovation in the scientific processes of product’s development, evaluation, manufacture, and use.[18] CPI has been defined as “the FDA’s national strategy for transforming the way FDA-regulated products i.e. human drugs, biological products, medical devices, veterinary drugs, foods, and cosmetics, are developed, evaluated, manufactured, and used”.[10]

The 2006 critical path opportunity list outlined specific key areas of critical path focus identified by FDA experts and the public.[7] These include:

- developing better evaluation tools like biomarkers and new assays
- streamlining clinical trials by modernizing the clinical trial sciences to make trials safe and efficient
- harnessing bioinformatics
- moving manufacturing into the 21^st^ century, using tools such as process analytic technology and nanotechnology
- developing products to address urgent public health needs including improved antimicrobial testing, new animal models to test bioterrorism countermeasures and vaccine testing
- focusing on at-risk populations, such as pediatrics

## Innovative Drug Development Tools of Critical Path Initiative

CPI promised to bring down cost and time of drug development by incorporating scientific innovations in the field of drug development without compromising efficacy and safety of the developed drugs. Various fields of drug development paradigm that were innovated or are still being innovated are development of biomarkers, investigational new drug (IND) phase and clinical trial designs.

### Developing new biomarkers

The FDA has created programs to foster use of biomarkers both for routine diagnostic and for drug development purposes.[19] First task as enlisted in FDA’s Critical Path Opportunity List 2006 is the development of new biomarkers to improve clinical trials and medical therapy. It is stressed to focus on the mapping of the process and criteria for qualifying biomarkers for use in product development.[7] Various disease and disorder specific biomarkers that have been suggested to be developed are:

- β-receptor polymorphism to predict long-term efficacy and safety in asthma
- troponin subtypes and inflammatory cytokines that reflect tissue damage or acute inflammation as circulating biomarkers in cardiovascular disease.
- Measurement of immune response to preventive vaccines as a marker of protection
- α-methylacyl CoA racemase expression as a predictor of disease progression in prostate cancer.

Need for development of novel biomarkers for systemic lupus erythematosus, inflammatory bowel disease, neuropsychiatric disorders, cancers, hepatitis C infection has also been emphasized, besides developing safety biomarkers and improving new imaging techniques to be used as biomarkers.[7] With the development of these biomarkers as standard and recognized surrogate endpoints, it will be pertinent to use these in documenting the efficacy and safety of new clinical entities in clinical trials. Moreover, these biomarkers will also be used as predictive tools for assessing the efficacy and outcome of drug treatment.

### Challenges in developing new biomarkers

Identifying biomarkers that are associated with clinical outcomes of a disease or a treatment given, is itself challenging. Still more troublesome is establishing a predictive model between relevant biomarkers and clinical outcomes in clinical development. Correlation between biomarker and true clinical endpoint definitely makes a prognostic marker but not a predictive biomarker. Prognostic markers can be used to separate good and poor-prognosis patients at the time of diagnosis. A predictive biomarker informs the treatment effect on the clinical endpoint. Thus, the difference has to be established, which is very difficult to achieve.[20]

Sometimes, it becomes difficult to ascertain which biomarker evidence is appropriate to guide the selection of patients for clinical testing. Moreover, it is very difficult to pinpoint the types and levels of evidence that are needed to accept a biomarker as a surrogate endpoint for product efficacy.[7] Above all, time and again, the potential and ability of surrogate endpoints as true predictive tools in clinical trials have been debated.[21,22]

### Progress in the field of biomarkers

The Predictive Safety Testing Consortium, a public-private partnership, led by the non-profit Critical Path Institute (C-Path), is facilitating the sharing of information by industry to develop new tools that can be qualified for use in drug development. This effort is taking place under the advice of FDA and the European Medicines Evaluation Agency (EMEA). In May 2008, FDA and EMEA announced that they had reviewed and accepted seven new biomarkers – laboratory tests on urine that signal kidney injury. These new tests can now be used in laboratory research to predict the safety of experimental drugs, enabling drugs to reach market faster and with greater confidence in their safety.[7]

Recently, Brain Natriuretic Peptide (BNP) has been proposed as a good biomarker for assessing prognosis and follow-up in pulmonary arterial hypertension.[23] Another example of development of new biomarker under the aegis of CPI is that of protein degradation fragments called neoepitopes which have proven useful for research on bone and cartilage and are collagen type I and collagen type II degradation products, respectively. These markers have utility in the translational approach, as they can be used to estimate safety and efficacy in both preclinical models and clinical settings.[24]

## Exploratory Investigational New Drug Studies/Microdosing Studies

Many compounds fail during different stages of drug development, and suboptimal pharmacokinetics (PKs) is stated to be a reason for failures. In traditional phase I studies, pharmacologic and toxicologic data collected from animal studies (pre-clinical studies) are submitted at the time of submission of new drug application (NDA). These animal studies are designed to permit the selection of a safe starting dose for humans, to estimate the margin of safety between a clinical and a toxic dose, and to predict PK and pharmacodynamic (PD) parameters. These early tests are usually resource intensive, requiring significant investment in product synthesis, animal use, laboratory analyses, and time. Many resources are invested on, and thus wasted, if candidate products are subsequently found to have unacceptable profiles on human evaluation. Less than 10% of applications for INDs for NME progress beyond the investigational stage to submission of NDA.[25]

To address this issue precisely, FDA released guidance for industry on exploratory IND (eIND) studies in 2006, commiserating with CPI.[25] The introduction of the eIND process allowed small, microdosing or phase 0 clinical trials to be conducted prior to traditional phase I trials, sometimes requiring considerably less chemistry, manufacturing and controls, or preclinical support.[26] Microdosing studies are designed to evaluate PKs or imaging of specific targets in a small number of study subjects and are designed not to induce pharmacologic effects. A microdose is defined as less than 1/100th of the dose of a test substance calculated (based on animal data) to yield a pharmacologic effect of the test substance with a maximum dose of ≤100 *µ*g (the maximum dose for protein products is ≤30 nmol).[27]

Thus, eIND differs from conventional IND in the sense that the NME are introduced firstly in humans in non-pharmacologic doses (phase 0) instead of conventional phase I studies, where NME is given in therapeutic doses. Determining PDs, PKs and metabolism of a drug at such an early stage in humans, by using phase 0 studies, will help in eliminating compounds that have sub-optimal properties and can aid in “go or no” decision of a compound; meaning development of a compound with sub-optimal properties can be stopped at this early stage itself, while one can go-ahead with compounds having optimal properties.. Also, due to the involvement of relatively smaller number of study subjects and use of limited number of doses of the study agent, the associated risk of toxicity of phase 0 is comparatively lower than traditional phase I studies. Therefore, requirement of toxicologic studies before starting phase 0 is less, enabling these studies to be initiated sooner than traditional phase I studies. These two factors will considerably reduce the cost and time of drug development.

### Challenges in conducting microdosing studies

For microdosing studies, ultrasensitive and specific analytical methods capable of measuring drugs and metabolites in pictogram range like accelerated mass spectrometer (AMS) are required. Moreover, drug compound must be radiolabeled.[28] The patients in a phase 0 trial have no hope of benefit from the drug; the dose is just too small. The small doses can give results that may not be relevant to the later real-world ones; both false positive and false negative results may be documented.[29] Another limitation is that some compounds dissolve readily at microdose, yielding good absorption characteristics; however, at therapeutic doses, they exhibit limited solubility, and absorption becomes dependent on the rate and extent of dissolution, which cannot be predicted at microdose levels.[27,30]

Another question of concern is whether microdosing predicts PK parameters accurately for drugs showing nonlinear kinetics. Many processes within the body involve the use of specialized transporters, enzymes and binding sites, which can be saturated so that the PK profile is very different at the higher therapeutic dose than that seen with the microdose.[27]

### Progress in the field of exploratory investigational new drug/microdosing studies

Using microdosing strategies, progress has been made in the field of oncology, where microdosing studies are of great help to determine the influx/efflux of IND to target human cells, thus rejecting useless compounds at an early stage of the drug development. As DNA damage is a critical step in cancer cell response to platinum (Pt) chemotherapy, it was hypothesized that low levels of Pt-induced DNA damage are predictive of chemoresistance and the same was verified by microdosing technique using AMS.[31]

With the application of microdosing strategies with environmental carcinogens to accelerate the evaluation and optimization of chemopreventive interventions, the cause of microdosing studies has been really boosted.[32] Recently, in Finland, Orion Pharma has completed phase 0 trial of investigational drugs ORM-14540 and ORM-12741, to know their PK profile using microdoses.[33] Over the last 10 years, Xceleron has developed a database which compares PK data at microdose and therapeutic dose levels, which shows that for 25 compounds studied, microdose PK scales to pharmacologic dose in over 80% of cases.[34]

## Adaptive Designed Clinical Trials

Unacceptable levels of attrition in the clinical stage of development are driving profound changes in the architecture, design, and analysis of clinical trials also. Moreover, the pharmaceutical industry is gradually coming to realize that the classically structured clinical trial does not offer enough flexibility to make use of continuously emerging knowledge that is generated as the trial progresses.[35] The purpose of adaptation in clinical trials is to give the investigator the flexibility for identifying the optimal clinical benefit of the test treatment under study without undermining the validity and integrity of the intended study.[36]

Adaptive design clinical trial is termed as “a study that includes a prospectively planned opportunity for modification of one or more specified aspects of the study design and hypotheses based on analysis of data (usually interim data) from subjects in the study”, according to the FDA’s new draft guidance for industry on adaptive design clinical trials, released in February 2010. Analyses of the accumulating study data are performed at pre-planned time-points within the study, with or without formal statistical hypothesis testing.[37] The term prospective here means that the adaptation was planned before data were examined in a un-blinded manner. Changes in study design, which were not prospectively planned, or study design aspects that are revised based on information obtained entirely from sources outside of the specific study are not considered adaptive design. Adaptation may be made to trial procedure and/or statistical procedure during the conduct of a clinical trial.[37]

Based on the adaptations employed, the commonly considered adaptive design methods in clinical trials include an adaptive randomization design, a group sequential design, a sample size re-estimation design, a drop-the-loser design, an adaptive dose finding design, a biomarker-adaptive design, an adaptive treatment-switching design, a hypothesis-adaptive design, an adaptive seamless phase II/III trial design, and a multiple adaptive design.[38]

An adaptive randomization design allows modification of randomization schedules based on varied or unequal probabilities of treatment assignment in order to increase the probability of success. A group sequential design allows for prematurely stopping a trial if there are safety or efficacy issues at the interim analysis, with options of additional adaptations. A sample size re-estimation design allows for sample size adjustment or re-estimation based on the observed data at interim, while a drop-the-loser design allows dropping the inferior treatment groups. An adaptive seamless phase II/III trial design addresses objectives that are normally achieved through separate trials in phase IIb and phase III of clinical development, within a single trial. This design uses data from patients enrolled before and after the adaptation in the final analysis. In a seamless adaptive design, phase II trial’s transitions into a phase III trial happens without pause, saving considerable drug development time.[38]

Prospect of these adaptive designed clinical trials are encouraging. Many compounds in drug development eventually fail, but these designs allow earlier detection and early stoppage of a clinical trial. Moreover, trial subjects are used more efficiently, and fewer subjects are given ineffective compounds, ineffective doses, or doses that are unnecessarily high. At the same time, pharmaceutical companies waste less on unsuccessful compounds and can more quickly re-assign resources to alternative drugs within their pipelines.

### Challenges in using adaptive designs in clinical trials

In practice, it is common to have three to five protocol amendments during the conduct of a clinical trial. One of the major impacts of many protocol amendments is that the target patient population may have been shifted during the process; as a result, the resultant actual patient population following certain modifications to the trial procedures may be a moving target patient population rather than a fixed target patient population, and consequently, the overall type I error rate may not be controlled.[39]

In addition, major adaptations of trial and/or statistical procedures of ongoing trials may result in a totally different trial that is unable to address the clinical questions the trial intends to answer. Moreover, significant adaptations may introduce bias/variation to data collection as the trial continues.[38]

There are also some operational difficulties with adaptive trials.[40] These trials commonly use Bayesian statistical approach, but this is computationally and logistically complex and might not be practically feasible in all situations. Quick and reliable electronic data collection would seem to be mandatory for a trial that is dependent on constant updating. Moreover, adaptive designs require computer-based simulations of clinical trials to develop the design, requiring more work-force.[41]

Validity and integrity of adaptive designed clinical trials from regulatory perspective is also of great concern.[42] Although FDA encourages use of adaptive designs, several regulatory concerns do exist. First, what level of adaptation will be acceptable to the regulatory agencies? Second, what are the regulatory standards for review and approval process of clinical data obtained from adaptive clinical trials with different levels of modifications? Third, has the clinical trial become a totally different clinical trial after the modifications, for addressing the study objectives of the originally planned clinical trial? These concerns should be addressed by the regulatory authorities before the adaptive design methods can be widely accepted in clinical research and development.[37]

### Progress in implementing adaptive designs in clinical trials

Regulatory agencies in the major markets have developed decisive positions on adaptive design clinical trials. Two-stage adaptive proof-of-concept and dose-finding adaptive designed clinical trials have already been used for migraine studies.[43] A 3 + 3 adaptive design is already in use in oncology studies.[44] In a 3 + 3 trial, three patients start at a given dose, and if no dose-limiting toxic effects are seen, three more patients are added to the trial at a higher dose. If there is one instance of limiting toxicity in the first group, three more patients are added at the same dose. If two (or all three) in any cohort show dose-limiting toxicity, the next lower dose is declared to be the maximum tolerated.

Recently, in March 2010, “Biomarkers Consortium” launched a pioneering multiagent adaptive clinical trial to treat breast cancer, intended to give several investigational drugs to treat breast cancer together at the same time, under a project named “Investigation of Serial Studies to Predict Your Therapeutic Response with Imaging And Molecular Analysis (I-SPY 2 TRIAL). The first agents expected to be tested include veliparib, conatumumab, figitumumab. In this trial, adaptive design will enable researchers to use early data from one set of patients to make decisions about patients later in the trial, and eliminate ineffective treatments more quickly.[45,46]

## Controversies in the Path of Critical Path Initiative

Under CPI, FDA is boosting the concept of partnerships with pharmaceutical houses and research organizations to accelerate the development of personalized medicines.[10] Point of concern here is if it is ethically and morally right on the part of FDA to have partnerships with some specific drug houses. Allegations of FDA being biased are bound to creep in, which may swell into a big controversy of FDA’s history, in the years to come.

Another question often raised is, if CPI has overstepped its domain and utility. Because globalization, rapidly evolving technologies, and emerging areas of science are having an increasing impact on all FDA-regulated products, CPI has gradually expanded its scope, leveraging the knowledge gained from new scientific fields to enhance the tools used in human and animal medicine and food safety.[10] Thus, it is pertinent to ask “Will FDA be able to manage so many affairs”.

Open-source, precompetitive research collaborations under the aegis of CPI are also a matter of concern. Many successful large industries, such as computer-chip manufacturers, have established successful precompetitive collaborations focusing on standards, applied science, and technology that advance the field for all stakeholders and benefit the public. But a 2010 study pointed out that the pharmaceutical industry has a well-earned reputation for fierce competition and does not demonstrate willingness to share data or knowledge.[47] With the launch of CPI, many pharmaceutical companies are also collaborating for precompetitive research.[48] But, questions have already been raised about the feasibility of these cross-company precompetitive collaborations and many pitfalls have already been noticed.[49,50] One obvious concern is the management of logistics for these collaborations. Another concern is the sharing of fruits from these cross-company open-source research collaborations and intellectual property rights over these collaborative researches. These conflicts may lead to unnecessary litigations, thus unduly increasing the cost and time of drug development, and undermining the ultimate goal of CPI.

## Progress in India on the Lines of Critical Path Initiative

Recently, ischemia modified albumin (IMA) has been reported to be a biomarker for myocardial ischemia/infarct, in a conference held in April 2010. IMA showed 100% sensitivity and 85.3% specificity in diagnosing patients with acute coronary syndrome and the results were comparable with those of cardiac troponin and CK-MB.[51]

In another novel effort of its kind, Indian researchers have reported Kat-G to be a specific and sensitive biomarker for the differential diagnosis of *Mycobacterium avium infection*. This protein was recognized as early as second week post-infection and the test can be developed into a simple serodiagnostic-based kit method. Results of this serodiagnostic technique were comparable to time-consuming biochemical tests, with the advantage that Kat-G based tests can be applied to HIV positive/AIDS patients who do not respond to T-cell based assays due to very low CD4^+^ T-cell counts.[52]

In another collaborative study, PK parameters after administering microdoses were compared with PK parameters after administering therapeutic doses. It was reported that for enalapril, losartan and atenolol, microdosing PK parameters were comparable with therapeutic dose values and showed linearity over therapeutic dose range.[53]

As reported in May 2010, Council of Scientific and Industrial Research (CSIR) of India has started an open-source drug discovery (OSDD) initiative, on the lines of FDA’s CPI. This program has provided a collaborative platform for scientists, doctors, engineers, technical experts, software professionals and others with a wide range of expertise, to enhance the drug discovery process. This project is focused on targeting tuberculosis, and is an internet-based project with no intellectual property. OSDD is now a strong community of 3200 participants from about 74 countries.[54]

## Conclusion

Present era is an era of global meltdown. Economy everywhere in the world has been hit by recession. Although pharmaceutical industry is a disease-driven industry and to some extent it has remained immune to global recession; shortage in inflow of funds due to economic crisis is showing its effects in the form of decline in the development of new drugs. Thus, innovative techniques as mentioned in CPI, which can bring down the escalating costs of drug development, are the need of the hour.

FDA is escalating its critical path work because a strong FDA is imperative to moving discoveries from microscope to market. The need for research in the barren field of critical path is not likely to end. Scientific advances will continue to create methodologic challenges in medicine that will require changes in the way we develop new products. But, a major question is sustainability, whether the CPI can maintain its momentum and substantively contribute to improved drug development.
